# Acceptance, Safety, and Effect Sizes in Online Dialectical Behavior Therapy for Borderline Personality Disorder: Interventional Pilot Study

**DOI:** 10.2196/66181

**Published:** 2025-01-14

**Authors:** Ruben Vonderlin, Tali Boritz, Carola Claus, Büsra Senyüz, Saskia Mahalingam, Rachel Tennenhouse, Stefanie Lis, Christian Schmahl, Jürgen Margraf, Tobias Teismann, Nikolaus Kleindienst, Shelley McMain, Martin Bohus

**Affiliations:** 1 Department of Psychosomatic Medicine and Psychotherapy Central Institute of Mental Health Medical Faculty Mannheim, Heidelberg University Mannheim Germany; 2 German Center for Mental Health (DZPG) Partner Site Mannheim - Heidelberg - Ulm Mannheim Germany; 3 Department of Psychology York University Toronto, ON Canada; 4 Centre for Addiction and Mental Health Borderline Personality Disorder Clinic Toronto, ON Canada; 5 Mental Health Research and Treatment Center Ruhr-University Bochum Bochum Germany; 6 Department of Clinical Psychology Central Institute of Mental Health Medical Faculty Mannheim, Heidelberg University Mannheim Germany; 7 Department of Psychiatry University of Toronto Toronto, ON Canada; 8 McLean Hospital Harvard Medical School Boston, MA United States

**Keywords:** dialectical behavior therapy, borderline personality disorder, online psychotherapy, virtual psychotherapy, telehealth, personality disorders, mental, psychotherapy, online, internet, telemedicine, psychiatry, psychiatric, acceptance

## Abstract

**Background:**

The potential of telehealth psychotherapy (ie, the online delivery of treatment via a video web-based platform) is gaining increased attention. However, there is skepticism about its acceptance, safety, and efficacy for patients with high emotional and behavioral dysregulation.

**Objective:**

This study aims to provide initial effect size estimates of symptom change from pre- to post treatment, and the acceptance and safety of telehealth dialectical behavior therapy (DBT) for individuals diagnosed with borderline personality disorder (BPD).

**Methods:**

A total of 39 individuals meeting the *DSM-5* (*Diagnostic and Statistical Manual of Mental Disorders* [Fifth Edition]) criteria for BPD received 1 year of outpatient telehealth DBT at 3 sites in Germany and Canada. Effect size estimates were assessed using pre-post measures of BPD symptoms, dissociation, and quality of life. Safety was evaluated by analyzing suicide attempts and self-harm. Additionally, acceptance and feasibility, satisfaction with treatment, useability of the telehealth format, and the quality of the therapeutic alliance were assessed from both therapists’ and patients’ perspectives. All analyses were conducted on both the intention-to-treat (ITT) and according-to-protocol (ATP) samples.

**Results:**

Analyses showed significant and large pre-post effect sizes for BPD symptoms (*d*=1.13 in the ITT sample and *d*=1.44 in the ATP sample; *P*<.001) and for quality of life (*d*=0.65 in the ITT sample and *d*=1.24 in the ATP sample). Dissociative symptoms showed small to nonsignificant reductions. Self-harm behaviors decreased significantly from 80% to 28% of all patients showing at least 1 self-harm behavior in the last 10 weeks (risk ratio 0.35). A high dropout rate of 38% was observed. One low-lethality suicide attempt was reported. Acceptance, feasibility, and satisfaction measures were high, although therapists reported only moderate useability of the telehealth format.

**Conclusions:**

Telehealth DBT for BPD showed large pre-post effect sizes for BPD symptoms and quality of life. While the telehealth format appeared feasible and well-accepted, the dropout rate was relatively high. Future research should compare the efficacy of telehealth DBT with in-person formats in randomized controlled trials. Overall, telehealth DBT might offer a potentially effective alternative treatment option, enhancing treatment accessibility. However, strategies for decreasing drop-out should be considered.

**Trial Registration:**

German Clinical Trials Register DRKS00027824; https://drks.de/search/en/trial/DRKS00027824

## Introduction

### Background

Borderline personality disorder (BPD) is a severe mental disorder characterized by high instability in affect, identity, and interpersonal relationships [1,2]. The prevalence of BPD is estimated to be between 0.7% and 2.7% in community samples [3,4] and between 11% and 35.6% in clinical samples [4]. BPD is associated with significant health care use and costs, as well as profound individual suffering and impaired quality of life [5-7].

According to international guidelines [8,9], psychotherapy is the first-line treatment for BPD. However, the complexity of symptoms such as high levels of suicidality, and interpersonal difficulties present challenges for psychological treatments [10,11]. Dialectical behavior therapy (DBT) [12-14] is a specialized treatment that was developed to support therapists in dealing with the complex symptom constellations that characterize BPD. DBT includes a dynamic hierarchy of treatment foci and a large set of therapeutic strategies and skills-based interventions to support individuals in building a life worth living. DBT is a multimodal psychological treatment that consists of individual and group therapy. A comprehensive DBT treatment includes both individual and group sessions that occur weekly over a 1-year period. DBT is an empirically supported treatment that has a robust research base demonstrating its efficacy in the treatment of BPD [15].

### The Potential Benefit of Telehealth DBT for BPD

While there are effective psychological treatments for BPD, DBT and other evidence-based treatments are difficult to access [16,17]. One significant barrier to accessing DBT is that given the complexity of the treatment (ie, its multiple components, length, and team-based model) the treatment is often localized in specialized treatment centers in urban settings; DBT-trained therapists and comprehensive DBT treatment programs are often not available for clients in remote or rural settings. This problem is even more pronounced in developing countries or low- and middle-income countries [18].

One solution for improving access to DBT is to expand the delivery options of the treatment to include telehealth treatment (ie, the online delivery of treatment via a video web-based platform). Propelled in large part by the COVID-19 pandemic, DBT practitioners around the world have already begun to shift to the telehealth delivery of the treatment, with many clinicians in both health care centers and in private practice currently offering telehealth DBT [19-21]. However, concerns have been raised regarding the efficacy, safety, and feasibility of telehealth-delivered psychotherapy, especially for patients with high levels of emotional and behavioral dysregulation. Although research has started to explore the benefits, barriers, and subjective experiences of clients and therapists engaged in telehealth DBT in routine care, to date there are no rigorous research trials that have examined the efficacy, safety, acceptance, and feasibility of telehealth DBT [19-29].

### Challenges of Telehealth Psychotherapy for BPD

Of particular concern is the limited ability of therapists to effectively deal with crisis situations and suicidal behavior in telehealth treatments [20,25,30]. Zalewski et al [28] conducted a qualitative study that shed light on various telehealth treatment challenges with patients with BPD, including patients missing sessions, turning off their cameras prematurely, logging off unexpectedly, or engaging in distracting behaviors like smoking or drinking alcohol during telehealth group therapy sessions. They also reported that some patients appeared dissociative during group sessions. Dissociation poses a particular challenge to the safety and efficacy of telehealth treatments for BPD. Previous research has underscored how dissociative experiences can lead to neurobiological alterations and impede emotional learning, thereby hindering successful psychotherapeutic outcomes [31-33]. Ideally, a therapist noticing dissociation during a session would intervene and provide guidance on how to use distress tolerance skills to decrease or interrupt dissociation. However, implementing these skills often involves strong sensory input or physical activity, which make be harder to facilitate telehealth versus in-person. More research is needed to investigate if and how dissociation and other therapy-interfering behaviors can be managed in telehealth treatments for BPD [34].

Another critical aspect of telehealth psychotherapy is the quality of the therapeutic alliance [20]. The interpersonal difficulties that are common in BPD, such as mistrust and rejection sensitivity, can make it harder to develop a strong alliance [35]. Previous DBT research suggests that a weaker therapeutic alliance is associated with higher therapy dropout rates [36] and that alliance rupture and repair processes in the early stage of treatment differ between recovered and unrecovered patients with BPD [37]. There is an ongoing debate as to whether the telehealth setting hampers the effective establishment of a therapeutic alliance and increases the likelihood of alliance ruptures. While some studies show comparability between telehealth and in-person treatment formats [38,39], others suggest that telehealth therapies may be less conducive to developing an alliance than in-person therapies. Despite the pivotal role of the therapeutic alliance in DBT, at this point, there is no conclusive evidence as to how well a therapeutic alliance can be established and maintained in telehealth DBT.

### Previous Research on Telehealth DBT

Two scoping reviews have been conducted to document the research evidence associated with the online delivery of DBT for individuals with BPD [24,40]. van Leeuwen et al [40] conducted their review to document the research evidence regarding the efficacy and clinical use of telepsychology in DBT. The studies they included in their review were heterogenous and ranged from the online delivery of treatment (eg, telehealth defined as videoconferencing for individual and group sessions, to the use of telephone coaching between sessions, to the integration of mobile apps as stand-alone or hybrid interventions, to the integration of virtual reality elements in treatment). Overall, they included 41 studies; however, only 2 of these trials investigated telehealth DBT applied by therapists via videoconferencing systems for patients with BPD specifically [41,42]. In a second review, Lakeman et al [24] similarly reviewed the online delivery of DBT for a range of different mental disorders. They showed that attendance tended to be higher in telehealth format and clinical improvements were comparable to in-person formats. However, many of the original trials also highlighted challenges related to risk management, therapist preparedness, and technical difficulties. The authors concluded that despite various challenges, the telehealth delivery of DBT programs is more accessible and feasible and equally acceptable, safe, and effective as the in-person delivery. However, of the 11 trials included in this review, only 1 trial was conducted with patients with BPD [43], therefore it remains unclear whether their conclusions can be generalized to BPD-specific populations.

While the aforementioned results suggest the telehealth delivery of DBT may be promising, a closer look into the primary studies included in the 2 reviews reveals a number of limitations that impact the conclusions we can draw from them. For example, Lopez et al [41] conducted a nonrandomized trial comparing attendance, client satisfaction, and group cohesion measures in a telehealth DBT skills group (n=20) compared to an in-person skills group (n=15). They found that attendance rates were significantly higher in the telehealth format (91% vs 75%), though there were no significant differences in satisfaction between the groups. Cohesion measures between group members and their therapists showed no differences, although cohesion between group members was significantly higher in the in-person group. Additionally, clients were not randomized but instead allowed to select their preferred group format. Finally, their study did not report on clinical outcomes related to borderline symptoms, which means we cannot compare the clinical effectiveness of the 2 treatment formats on this domain.

Salamin et al [42] analyzed diary card data of 7 patients with BPD within a treatment that had to be shifted from in-person to telehealth format due to the COVID-19 pandemic. In the first 8 weeks of treatment, patients received weekly in-person individual sessions as well as group sessions. Due to COVID-19 restrictions, treatment shifted to telehealth, and patients received weekly individual sessions by telephone or videoconferencing and group therapy was replaced with personal coaching. Weekly monitoring of problem behaviors showed a decrease in binge-eating behaviors and a trend toward a decrease in alcohol consumption following the shift to telehealth treatment. Results also indicated that while clients reported a decrease in feelings of shame or guilt, fear and tension following the move to a telehealth format, their general level of distress increased. Given the small sample size and lack of a control group, as well as the significant confound of the start of the COVID-19 pandemic that spurred the change in format to begin with, results from this study cannot reliably be attributed to the treatment or delivery format.

Alavi et al [43] investigated the effectiveness of an email-based DBT intervention compared to an in-person DBT skills group in 107 patients with BPD. While there were no significant differences between the treatment formats, these findings must be interpreted with caution: the DBT intervention consisted of only 15 sessions, and less than 50% of patients in either group completed treatment and were included in the analysis.

In summary, there is limited evidence regarding the feasibility and effectiveness of telehealth comprehensive DBT.

### Objective of This Study

In this study, we aimed to investigate the acceptance, safety, and effect sizes of a 1-year comprehensive outpatient DBT program delivered telehealth for individuals with BPD within a controlled research setting. We were interested in the effect sizes for BPD symptoms and quality of life, and whether self-harm and dissociative symptoms could be targeted and effectively managed in a telehealth setting. To understand the acceptability and feasibility of telehealth DBT, we collected clients’ and therapists’ perspectives on the delivery of telehealth treatment. Finally, to determine whether a strong therapeutic relationship could be established in a telehealth treatment, we measured both therapists’ and clients’ ratings of the quality of the alliance throughout treatment.

## Methods

### Study Design

The data originate from a pilot psychotherapy trial comparing 2 different forms of DBT (standard DBT and trauma-focused DBT) within a telehealth setting. This pilot trial was designed to test the feasibility, safety, and acceptance of telehealth DBT and to prepare for a larger randomized controlled trial (RCT) of trauma-focused DBT. In order to ensure comparability with existing studies in the field, we chose to solely analyze the study arm that delivered telehealth standard (comprehensive) DBT, as developed by Marsha Linehan [12-14]. The study was conducted across 2 sites in Germany (Mannheim and Bochum) and 1 site in Canada (Toronto) between February 2022 and June 2023. The pilot study was registered in the German Clinical Trials Register (DRKS00027824) and received approval from the respective ethical review boards at each participating site.

### Intervention and Treatment

Participants received a 12-month outpatient standard DBT intervention, which comprised weekly individual therapy sessions (each lasting 50 minutes) and weekly group sessions (each lasting 100 minutes). All individual and group sessions were delivered via telehealth using videoconferencing platforms. The treatment was based on Marsha Linehan DBT treatment manual [13,14]. The group therapy focused on skill-building in mindfulness, distress tolerance, emotion regulation, and interpersonal effectiveness. Individual session agendas were informed by a review of clients’ diary cards. All skills were taught twice (ie, 2 rounds of a 20-session curriculum). In individual therapy, treatment targets were prioritized based on the dynamic hierarchy focus of DBT that addresses life-threatening behavior, therapy-interfering behavior, and quality-of-life-interfering behavior, as needed. After completing the 12-month therapy period, clients were offered up to 5 booster sessions, which could be scheduled flexibly within the 6 months following treatment termination. Treatment was considered concluded once the booster sessions were finished. No specific adjustments were made to the treatment content to accommodate the telehealth format. However, patients completed their diary cards electronically via an app on their personal smartphones to facilitate diary card review. Participants who missed 4 consecutive weeks of individual therapy were categorized as treatment dropouts. Completion according to protocol (ATP) was defined as attending a minimum of 32 individual sessions, which equaled 80% of the expected dosage. All therapists (n=16) were trained in DBT with at least 2 days of theoretical DBT training completed at the beginning of the study (mean 4.7, SD 2.2 days), and at least half a year of clinical DBT experience (mean 3.6, SD 3.4 years). All therapists participated in a DBT consultation team to guide the treatment, which occurred on a weekly basis, consisting of 1.5-hour sessions per week.

### Participants

Participants were recruited from outpatient clinic waiting lists in Mannheim, Bochum, and Toronto, as well as through advertisements and therapist referrals. Inclusion criteria were individuals aged 18-65 years with a diagnosis of BPD, and meeting at least 5 of 9 criteria outlined in the *DSM-5* (*Diagnostic and Statistical Manual of Mental Disorders* [Fifth Edition]). Additionally, participants needed to commit to 1 year of outpatient treatment and be willing to engage in telehealth therapy (with internet-enabled devices provided to participants, if needed). Proficiency in English (for the Toronto site) or German (for the Mannheim and Bochum sites) and written informed consent were further prerequisites for participation. Exclusion criteria represented symptoms that indicated a different treatment model or setting was likely needed, such as a lifetime diagnosis of schizophrenia, bipolar I disorder, intellectual disability (IQ<70), posttraumatic stress disorder (PTSD), a BMI below 17.5, severe substance use disorder according to *DSM-5* requiring medical withdrawal support, and dementia. Additionally, individuals with more than 8 weeks of DBT in the preceding year were excluded.

**Figure 1:**
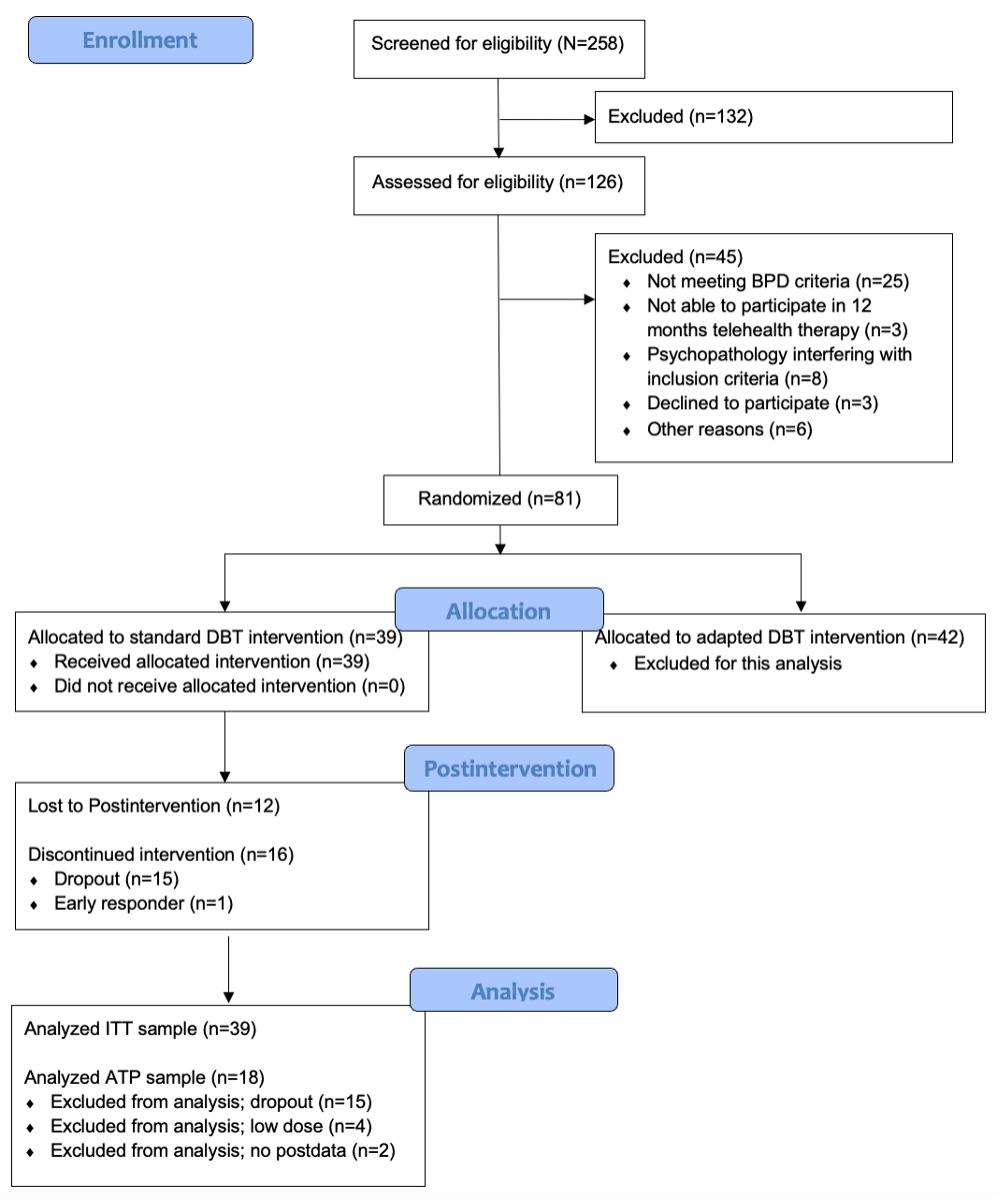
The flowchart shows the progression of participants throughout the study. ATP: according to protocol; BPD: borderline personality disorder; DBT: dialectical behavior therapy; ITT: intention-to-treat.

As depicted in the flowchart (Figure 1), 258 individuals underwent telephone screening for eligibility, with 126 undergoing full assessment. Ultimately, 81 (64%) individuals met the inclusion or exclusion criteria and were randomized to receive either standard DBT treatment (n=39; 48%) or a newly adapted DBT treatment (trauma-focused DBT; n=42, 52%). As this study only analyzed acceptance, safety, and effect sizes for standard telehealth DBT, our final sample comprised 39 participants enrolled in the 1-year telehealth standard DBT program. Among these, 15 (38%) individuals discontinued treatment and were classified as dropouts. One client ended the treatment as an early responder. Additionally, 12 (31%) participants did not provide assessments postintervention and were excluded from the analysis ATP. Consequently, 18 (46%) participants were included in the ATP analysis post intervention. The intent-to-treat (ITT) sample comprised all 39 participants who were randomly assigned to telehealth standard DBT.

### Measures

#### Primary Clinical Outcome

##### Borderline Symptom List-23

The Borderline Symptom List-23 (BSL-23) [44] was used to assess the borderline symptoms of participants throughout the trial. This self-report instrument comprises 23 items assessing borderline-typical symptoms on a 5-point Likert Scale, ranging from 0=not at all to 4=very strong. Widely used, the BSL-23 has demonstrated good psychometric properties [44,45]. In our sample, Cronbach α for the BSL-23 was 0.92. Assessment points included pre- and postintervention points, as well as every 3 months during the treatment period.

#### Secondary Clinical Outcomes

##### Deliberate Self-Harm Inventory

The Deliberate Self-Harm Inventory (DSHI) [46] is a self-report questionnaire designed to assess nonsuicidal self-injuries (NSSIs). Comprising 17 items, the DSHI captures data on the frequency, severity, duration, and type of self-harm behaviors. This instrument was administered both before and post intervention, as well as every 3 months during the treatment phase. In these analyses, we used the frequency score of the DSHI to determine the proportion of clients showing any NSSI in the last 10 weeks.

##### Dissociative Symptom Scale

The Dissociative Symptom Scale (DSS) [47] is a 20-item self-report questionnaire aimed at assessing dissociative symptoms. Participants are asked how often they have experienced dissociative symptoms over the last week ranging from 0=not at all to 4=more than once a day. It consists of 4 dimensions: depersonalization, derealization, gaps in awareness or memory, and dissociative reexperiencing. Since we were interested in the effect sizes on dissociative symptoms as a secondary outcome, we analyzed a global mean of the DSS across all dimensions. The DSS was administered before and post interventions as well as every 3 months during treatment.

#### Feasibility and Acceptability Outcomes

##### Acceptability of Intervention Measure

The Acceptability of Intervention Measure (AIM) [48] is a 15-item scale designed to assess therapists’ perspectives on the acceptability, feasibility, and appropriateness of the delivered treatment. Widely used in implementation research, AIM has exhibited robust psychometric properties across various studies [48]. In our sample, intercorrelations between dimensions ranged from *r*=0.81 to 0.90. Therefore, we computed a mean score by combining the 3 dimensions for analysis. Administration occurred after 6 months of treatment and post intervention.

##### Client Satisfaction Questionnaire

The Client Satisfaction Questionnaire (CSQ-8) [49] is an 8-item scale that measures the client’s satisfaction with the treatment and treatment provider on a 4-point Likert scale. The CSQ-8 was administered after 6 months of treatment as well as post intervention.

##### Usability of Technology

The unified theory of acceptance and use of technology (UTAUT) [50] assesses acceptance toward digital health care interventions based on the UTAUT framework. The questionnaire consists of 20 items assessing behavioral intention (4 items), performance expectancy (3 items), effort expectancy (3 items), social influence (2 items), facilitating conditions (2 items), internet anxiety (3 items), and experience with the internet (3 items) on a 5-point Likert scale ranging from 0=not at all to 4=very strong. We adapted items to also assess their usability throughout the treatment. The UTAUT was administered before and post interventions as well as at 6 months (midtreatment). Intercorrelations between dimensions ranged from *r*=0.80 to 0.91. Consequently, we computed a mean score to combine dimensions for analysis.

##### Severe Adverse Events

Severe adverse events (SAEs) were defined in accordance with the guidelines of good clinical practice. They encompassed suicide attempts or other events leading to death or posing an acute threat to life, where the study participant is in imminent danger of death at the time of the SAE. Therapists were queried weekly regarding the occurrence of potential SAEs. In the event of an SAE, therapists provided additional details to assess its intensity, outcome, and relationship to psychotherapeutic treatment. For safety purposes, all observed SAEs were promptly reported to the responsible ethics committee at the respective study site.

##### Working Alliance Inventory

The Working Alliance Inventory (WAI) [51] was used to assess the therapeutic alliance from both therapists’ and clients’ perspectives. The scale comprises 12 items assessing the therapeutic alliance on 3 dimensions: bonding, agreement on tasks, and agreement on goals. Therapists and their clients rate these items on a 7-point Likert scale ranging from 1=never to 7=always. Each therapist received separate questionnaires for each of their clients if they treated more than 1 client. The WAI was administered every 3 months during the treatment period. In our sample, intercorrelations between dimensions ranged from *r*=0.81 to 0.87 on the client level and from *r*=0.80 to 0.90 on the therapist level. Consequently, we computed a mean score to combine the 3 dimensions for analysis.

### Statistical Analyses

To determine treatment effects, we analyzed BPD symptoms on the BSL-23 as the primary outcome. As secondary outcomes, we analyzed the quality of life, dissociation, and self-harm of participants throughout the treatment. Given the nonlinearity of treatment effects over time, we used a nonparametric Friedman test to assess differences among repeated measures. The Friedman test is robust and capable of handling nonnormal distributions and outliers. Effect sizes for pre-post comparisons for each outcome were analyzed using Cohen *d*, providing insights into the magnitude of treatment effects. We also calculated 95% CIs to test the robustness of effect size estimates. To compare the proportion of patients showing at least 1 NSSI in the last 10 weeks between pre- and posttreatments, we calculated risk ratio (RR) according to Altman [52] with the following equation, where *a* represented the number of patients with at least 1 NSSI at postintervention, *b* represented the number of patients without NSSI at postintervention, *c* represented the number of patients with at least 1 NSSI at preintervention, and *d* represented the number of patients without self-harm at preintervention.



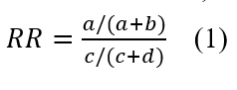



To assess the safety of the telehealth trial, we examined SAEs (eg, serious and life-threatening suicide attempts) throughout the study, NSSI as well as individual symptom trajectories of clients on the BSL-23 to identify potential symptom deterioration throughout the treatment.

To evaluate the feasibility and acceptance of the telehealth trial, we conducted descriptive analyses of the client and therapist feasibility and acceptance measures, as well as client and therapist ratings of the therapeutic alliance.

To analyze the intention-to-treat (ITT) sample, including all participants who had been randomized to the treatment, we imputed missing data by multiple imputation procedures [53]. Multiple imputation was based on the scale-level values of other assessment points and the dropout status (yes or no) to impute missing data. Since the missing pattern was not monotone, the Markov Chain Monte Carlo method [54] was used. Multiple imputation was based on the SAS (version 9.4; SAS Institute) multiple imputation procedures (1000 runs) and MIANALYZE. *P* values<.05 (2-tailed) were considered statistically significant.

### Ethical Considerations

This study protocol was reviewed and approved by the applicable ethics committees at each study center, Medical Faculty Mannheim at Heidelberg University in Mannheim of the leading committee for all German sites including Ruhr University Bochum (2021-628-MA), and Center for Addiction and Mental Health, Toronto, Ontario, Canada (023-2023). A written informed consent was obtained from all participants in the study. At the start of the trial, all participants were assigned a randomly generated study code, ensuring that all data were processed and analyzed in a pseudonymized format. Participants did not receive financial compensation for data assessments conducted during the treatment. However, those who discontinued the treatment prematurely were compensated €20 (US $21) for each subsequent assessment time point. At the Canadian study site in Toronto, all clients received CAD $15 (US $10.5) per assessment hour.

## Results

### Participants

The mean age of participants was 31.1 (SD 10.6) years, with 76.9% (n=30) born female. On average, participants met 6.18 (SD 1.07) criteria for BPD according to *DSM-5*. Additionally, 74.4% of participants had a comorbid affective disorder, 53.8% had a comorbid anxiety disorder, and 23.1% (n=9) had a comorbid eating disorder. Psychotropic medication was received by 97.5% of clients. Table 1 presents the demographic characteristics of the sample.

**Table 1:** Demographic and clinical characteristics of participants.

Baseline characteristics			Full Sample	
			Values	Range
Age (years), mean (SD)			31.1 (10.6)	19-58
Sex (female), n (%)			30 (76.9)	N/A^a^
**Highest educational level, n (%)**				
		No graduation	0 (0)	N/A
		Lower secondary school (hauptschule)	6 (15.4)	N/A
		Intermediate secondary school (realschule)	11 (28.2)	N/A
		Higher secondary school (abitur)	13 (33.3)	N/A
		University or postgraduate degree	9 (23)	N/A
**Marital status, n (%)**				
	Single		26 (66.7)	N/A
	Married or partnered		8 (20.5)	N/A
	Divorced or widowed		5 (12.8)	N/A
**Employment, n (%)**				
		Unemployed	5 (12.8)	N/A
		Student	4 (10.3)	N/A
		Employed	28 (71.8)	N/A
		Other^b^	2 (5.1)	N/A
BPD^c^ criteria (IPDE^d^), mean (SD)			6.18 (1.07)	5-8
**Comorbidities**^e^, **n (%)**				
		Affective disorders	29 (74.4)	N/A
		Anxiety disorders	18 (46.2)	N/A
		Eating disorders	9 (23.1)	N/A
		Obsessive compulsive disorder	5 (12.8)	N/A
**Psychotropic medications, n (%)**				
		Any psychotropic medication	31 (79.5)	N/A
		Antidepressants	22 (56.4)	N/A
		Neuroleptics	14 (35.9)	N/A
		Mood stabilizers	1 (2.6)	N/A
		Benzodiazepines	2 (5.1)	N/A
		Others	16 (41)	N/A
1 and more suicide attempt (lifetime), n (%)			21 (54)	N/A
1 and more nonsuicidal self-injury in the last month, n (%)			31 (80)	N/A

^a^N/A: not applicable.

^b^n=1 identified as part-time student, n=1 reported current reintegration in the labor market.

^c^BPD: borderline personality disorder.

^d^IPDE: International Personality Disorder Examination.

^e^Comorbidities were categorized as follows: affective disorders (depressive disorder and bipolar II disorder); anxiety disorders (panic disorder, agoraphobia, social anxiety disorder and generalized anxiety disorder); and eating disorders (anorexia, bulimia, and binge eating).

### Effect Size Estimations

**Figure 2:**
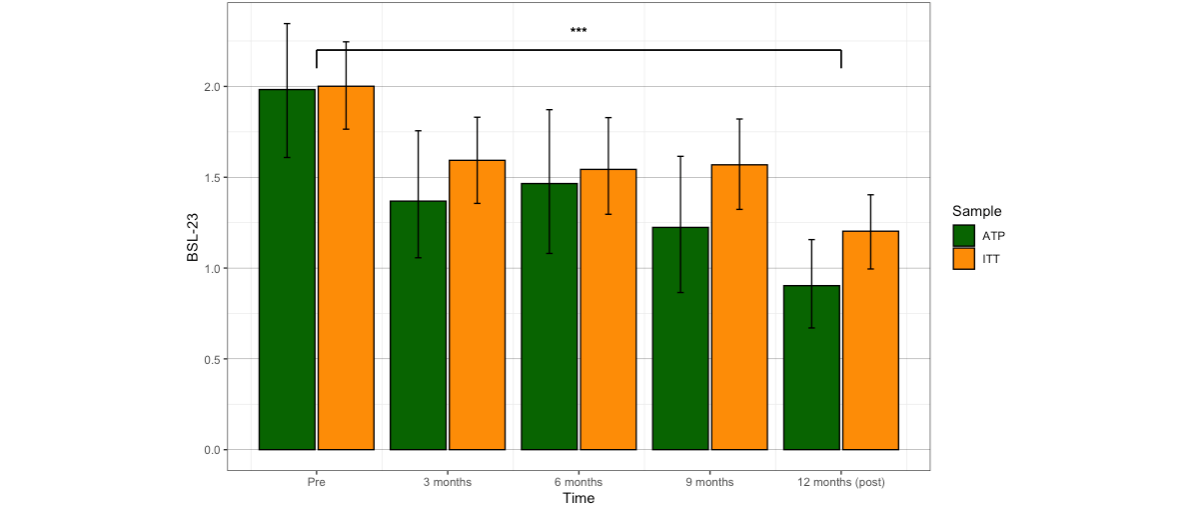
Course of borderline symptoms as the primary outcome in the ITT sample (n=39) as well as the ATP sample (n=18). Error bars represent 95% CIs using the bootstrapping method. ATP: according to protocol; BSL: Borderline Symptom List; ITT: intention-to-treat.

**Figure 3:**
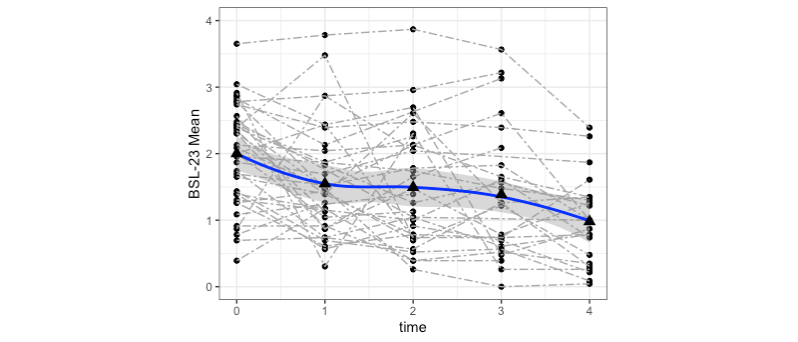
Individual data of participants’ borderline symptoms over the course of the whole treatment. BSL: Borderline Symptom List-23.

The effect size estimations revealed a significant decrease in BPD symptoms (BSL-23), our primary outcome, with large effect sizes observed in both the ATP (*χ²*_4_=24.9; *P*<.001; *d*=1.44) and the ITT sample (*χ²*_4_=38.8; *P*<.001; *d*=1.04; Figure 2). As illustrated in Figure 3, the most substantial reduction in BPD symptoms occurred within the first 3 months of treatment, with a further albeit smaller decrease observed throughout the remainder of the treatment period. Post–BSL-23 scores were a mean 0.91 (SD 0.60) in the ATP sample and a mean 1.20 (SD 0.65) in the ITT sample, indicating mild BPD symptoms in the ATP sample and moderate BPD symptoms in the ITT sample after treatment, respectively.

Dissociative symptoms exhibited a small, but significant decline in the ATP (*χ²*_4_=11.1; *P*=.02; *d*=0.37) and ITT samples (*χ²*_4_=12.3; *P*=.02; *d*=0.45) with clients reporting fewer dissociative symptoms throughout the treatment course. However, all 95% CIs of dissociation effect sizes included zero. Self-harm (DSHI scores) significantly decreased over the course of treatment, with 80% of clients engaging in at least 1 self-harm event in the last 10 weeks before treatment compared to 28% after treatment (*χ²*_4_=16.60; *P*<.001; *RR* 0.35).

The significant reduction in clinical symptoms was mirrored by an increase in quality of life, displaying a large effect size in the ATP (*χ²*_4_=14.5; *P*<.001; *d*=1.24) and a medium effect size in the ITT sample (χ²_4_= 31.0; *P*<.001; *d*=0.65). The pre-post changes of borderline symptoms and quality of life were significantly intercorrelated by *r*=–0.55 (t_37_=–4.04; *P*<.001). Detailed results are presented in Table 2.

**Table 2:** Mean, SD, Friedman Test, and effect size statistics for study variables for ITT^a^ and ATP^b^ samples^c^.

Variable				Baseline, mean (SD)		3 months, mean (SD)	6 months, mean (SD)	9 months, mean (SD)	12 months, mean (SD)	Friedman test		Effect size *d* (pre-post 95% CI)
										Chi-square (*df*)	*P* value	
**Effectiveness measures**												
	**ITT (*n*=39)**											
		Borderline symptoms (BSL-23^d^)	2.00 (0.76)		1.59 (0.78)		1.54 (0.84)	1.57 (0.86)	1.20 (0.65)	38.8 (4)	<.001	1.13 (0.64-1.61)
		Quality of life (ReQoL^e^)	1.48 (0.60)		1.83 (0.55)		2.01 (0.74)	1.71 (0.67)	1.93 (0.76)	31.0 (4)	<.001	0.65 (1.12-0.19)
		Dissociation (DSS^f^)	0.91 (0.61)		0.81 (0.59)		0.80 (0.68)	0.79 (0.44)	0.65 (0.55)	12.3 (4)	.015	0.45 (–0.01 to 0.90)
	**Per Protocol (ATP; n=18)**											
		Borderline symptoms (BSL-23)	1.98 (0.85)		1.38 (0.80)		1.47 (0.96)	1.22 (0.87)	0.91 (0.60)	24.9 (4)	<.001	1.44 (0.70-2.17)
		Quality of life (ReQoL)	1.55 (0.58)		1.93 (0.48)		2.15 (0.81)	2.05 (0.65)	2.33 (0.68)	14.5 (4)	<.001	1.24 (0.64-1.61)
		Dissociation (DSS)	0.99 (0.67)		0.83 (0.64)		0.88 (0.86)	0.68 (0.50)	0.72 (0.75)	11.1 (4)	.023	0.37 (–0.29 to 1.04)
		NSSI^g^ (%; DSHI^h^)	0.80 (0.41)		0.68 (0.48)		0.40 (0.50)	0.42 (0.50)	0.28 (0.46)	16.6 (4)	<.001	0.35 (RR^i^)
**Acceptance Measures**												
	**Client measures**											
		Client satisfaction (1-4; CSQ^j^)	—^k^		—		3.33 (0.54)	—	3.40 (0.74)	—	—	—
		Useability of technology (0-4; UTAUT^l^)	2.81 (0.59)		—		2.83 (0.72)	—	3.13 (0.67)	—	—	—
		Therapeutic alliance (1-7; WAI^m^)	—		5.52 (1.08)		5.89 (0.75)	5.65 (1.28)	—	—	—	—
	**Therapist measures**											
		Acceptance (1-5; AIM^n^)	—		—		4.21 (0.74)	—	4.53 (0.61)	—	—	—
		Useability of technology (0-4; UTAUT)	2.48 (0.84)		—		—	—	2.57 (0.71)	—	—	—
		Therapeutic alliance (1-7; WAI)	—		5.24 (0.98)		5.30 (0.99)	5.40 (0.88)	—	—	—	—

^a^ITT: intention-to-treat.

^b^ATP: according-to-protocol.

^c^Effect Sizes for continuous data were calculated using Cohen *d*. Effect sizes for categorical data (ie, NSSI) were calculated using risk ratio estimates.

^d^BSL-23: Borderline Symptom List-23.

^e^ReQoL: recovering quality of life.

^f^DSS: Dissociative Symptom Scale.

^g^NSSI: nonsuicidal self-injury.

^h^DSHI: Deliberate Self-Harm Inventory.

^i^RR: risk ratio

^j^CSQ: Client Satisfaction Questionnaire.

^k^Not applicable because variable was not assessed at this time-point

^l^UTAUT: unified theory of acceptance and use of technology.

^m^WAI: Working Alliance Inventory.

^n^AIM: Acceptability of Intervention Measure.

### Safety

During the course of treatment, 1 SAE was recorded. One patient was hospitalized following a suicide attempt involving an overdose in week 12 of treatment. The therapist rated this SAE as moderate and potentially linked to the psychotherapeutic treatment. In addition, individual patient trajectories in BPD symptoms were observed graphically for safety assessment, as illustrated in Figure 3. The majority of clients demonstrated a consistent decrease in BPD symptoms throughout the treatment duration.

### Acceptance, Feasibility, and Usability of Telehealth Treatment

The results regarding the acceptance and feasibility of clients are summarized in Table 2 and depicted in Multimedia Appendix 1. Clients reported high levels of satisfaction with the treatment. 97% (29/30) of clients reported high satisfaction with telehealth DBT treatment midway through therapy (mean 3.33, SD 0.54 on the CSQ-8) and 87% (20/23) reported high satisfaction at the end of treatment (mean 3.40, SD 0.74). Prior to treatment, 69% (27/39) of clients anticipated that the telehealth format would be very or mostly usable for receiving DBT treatment (mean 2.81, SD 0.59 on the UTAUT). This percentage increased slightly during treatment to 77% (23/30) at midtherapy (mean 2.83, SD 0.72) and further to 83% (19/23) at the end of treatment (mean 3.13, SD 0.67).

The results regarding the acceptance and feasibility of therapists are summarized in Table 2 and depicted in Multimedia Appendix 2. From therapists' perspective, 86% (12/14) found the telehealth DBT treatment to be very acceptable, feasible, and appropriate for their clients (mean 4.21, SD 0.74 on the AIM). This percentage rose to 91% (10/11) after the completion of treatment (mean 4.53, SD 0.61). Prior to treatment, 53% (8/15) of therapists anticipated that the telehealth format would be very much or mostly usable for delivering DBT treatment (mean 2.48, SD 0.84 on the UTAUT). However, this percentage slightly decreased during treatment to 45% (5/11), with therapists rating the telehealth format as very or mostly usable for delivering DBT after treatment (mean 2.57, SD 0.71).

### Therapeutic Alliance

The therapeutic alliance, as assessed by both clients and therapists, is summarized in Table 2 and depicted in Multimedia Appendix 1 for clients and Multimedia Appendix 2 for therapists. From the client perspective, 82% (28/34) reported experiencing a (very) high affective bond with their therapists and demonstrated (very) high agreement on treatment-related tasks and goals (mean 5.52, SD 1.08 on the WAI). This percentage increased to 96% (27/28) at midtherapy (mean 5.89, SD 0.75) before slightly decreasing to 84% at 9 months into treatment (mean 5.65, SD 1.28).

Similarly, therapist perspectives revealed that 80% (28/25) reported a (very) high affective bond with their clients and rated (very) high agreement on treatment-related tasks and goals (mean 5.24, SD 0.98 on the WAI). This percentage increased slightly to 87% (16/19) at midtherapy (mean 5.30, SD 0.99) before decreasing slightly to 84% (21/25) at 9 months into treatment (mean 5.40, SD 0.88).

A moderate yet significant intercorrelation was observed between therapist and client ratings, with *r*=0.52 (t_78_=5.35; *P*<.001) across all subscales and all 3 assessment times.

## Discussion

### Primary Results

This pilot study aimed to provide initial insights into the acceptance, safety, and effect sizes of standard (comprehensive) DBT treatment delivered via telehealth (ie, the online delivery of treatment via a video web-based platform) within a controlled research setting. Our findings indicate large pre-post effect sizes on BPD symptoms (*d*=1.13-1.44) and quality of life (*d*=0.65-1.24) in both the ITT and ATP samples, despite a relatively high dropout rate of 38% (n=15). One SAE was recorded during the study, attributed to hospitalization following a suicide attempt. Client ratings reflected high levels of satisfaction and usability, with 97% (29/30) expressing high satisfaction with the treatment and 83% (19/23) reporting high usability of the telehealth format. Therapists also expressed strong acceptance of the treatment, with 91% (10/11) indicating high acceptance. Interestingly, therapists rated the usability of the telehealth format comparatively lower than clients, with only 53% (8/15) reporting high usability. The majority of clients (27/28, 96%) and therapists (16/19, 87%) were able to establish a strong therapeutic relationship through telehealth contact.

### Comparison With Prior DBT Studies

The dropout rate of 38% (n=15) in this telehealth trial was higher than the average dropout rate of 28% reported in the meta-analysis on in-person DBT trials [55]. One possible explanation for this is the influence of the telehealth setting. Given both the heterogeneity in dropout rates across psychotherapy trials and the lack of an in-person control group in this study to contextualize this result, this finding should be interpreted with caution. Past research has yielded conflicting results regarding attendance and dropout rates in telehealth versus in-person treatments, with some studies indicating no differences between telehealth and in-person DBT, while others have suggested even higher attendance rates in the telehealth format [26,41]. Future studies should aim to directly compare dropout rates between telehealth and in-person DBT programs within RCTs as a broader framework is necessary to determine which clients are more likely to benefit from telehealth settings.

The estimation of effect sizes in this trial demonstrated a significant reduction in BPD symptoms, with large effect sizes of *d*=1.13 in the ITT sample and *d*=1.44 in the ATP sample throughout the treatment period. According to the interpretation guidelines for the BSL-23 proposed by Kleindienst et al [56], participants experienced a decrease in average borderline symptom severity from high at baseline to moderate at post-intervention in the ITT sample, and mild in the ATP sample, respectively. Comparing these outcomes with previous in-person DBT trials using a similar treatment dosage, our results suggest that clinical effectiveness in our trial is comparable or even slightly higher. For instance, McMain et al [57] reported a pre-post reduction of *d*=0.67. Similarly, Priebe et al [58] observed a significant decrease in BPD symptoms, as assessed by the Zanarini Rating Scale, with an effect size of *d*=0.71 from pre- to postintervention in a 12-month in-person DBT treatment. McMain et al [59] found that a 12-month in-person DBT treatment yielded a pre-post effect size of *d*=1.23 on the Zanarini Rating Scale. Sinnaeve et al [60] demonstrated a significant decrease in the Borderline Personality Disorder Symptom Index [61], with an effect size of *d*=1.15 from pre- to post-intervention in a 12-month outpatient in-person DBT treatment.

Of note, in our study, dissociative symptoms showed only small effect sizes of *d*=0.37 in the ATP and *d*=0.45 in the ITT sample with the 95% CIs containing 0. This finding may be due, in part, to the relatively low baseline dissociative scores of mean 0.91 (SD 0.61) and mean 0.99 (SD 0.67) likely related to our exclusion of patients with BPD with comorbid PTSD, who typically present higher dissociative symptoms compared to those without PTSD [62]. Our dissociation findings therefore continue the ongoing debate on the effectiveness of DBT in reducing dissociative experiences. According to the current Cochrane Review, DBT has a medium effect size for dissociation (*d*=0.43) across primary studies compared to waitlist and treatment-as-usual control groups [15]. Moreover, research suggests that greater effects on dissociation can be achieved by adapting a trauma-focused approach within DBT and explicitly teaching clients antidissociative skills, which were not included in our standard DBT protocol [63,64]. Consequently, our study does not allow us to conclude whether and how dissociative symptoms can be effectively addressed through the telehealth format. Future research should address this question by directly comparing the management of dissociative symptoms in telehealth versus in-person settings.

Self-harm and suicidal behavior in our study significantly decreased with a large reduction of participants’ NSSI of RR 0.35 in the ATP sample. While 80% of participants reported engaging in at least 1 self-harm episode in the months preceding treatment initiation, this number decreased significantly to 28% postintervention. This finding aligns with prior research indicating the efficacy of DBT in reducing NSSI behaviors by empowering individuals to attain behavioral control [60,65,66]. Importantly, our study suggests that the reduction of self-harm can be also effectively achieved in a telehealth format. Notably, there was 1 SAE, a suicide attempt by overdose, during the treatment period, with no recorded completed suicides. In previous in-person DBT trials, the prevalence of suicide attempts requiring medical attention (eg, emergency department visits) over a 12-month treatment period has ranged from 3.5% to 6.7% [57,67,68]. Based on these prevalence rates, we would expect 1 to 3 suicide attempts in a cohort of 39 patients. Thus, the single suicide attempt recorded in our study aligns with this expected range and does not suggest an increased occurrence of suicide attempts in telehealth treatment settings.

In terms of the therapeutic alliance, our study showed that over 80% of clients and therapists reported a very strong alliance. Mean scores for the therapeutic alliance ranged from mean 5.24 (SD 0.98) to mean 5.89 (SD 0.75) on a 7-point Likert scale of the WAI. These scores are comparable to, or even higher than, those reported in studies administering the same questionnaire in in-person DBT trials [36,69]. However, this finding may be biased as participants who dropped out of treatment also discontinued completing the alliance measure, potentially inflating alliance ratings over the course of treatment. Nonetheless, our results indicate that a strong therapeutic alliance can be achieved in telehealth DBT settings. This finding challenges the argument that telehealth treatment leads to heightened disengagement or weak therapeutic alliances between clients and therapists [20,70].

Satisfaction and acceptance ratings in our sample were high, with 91% of therapists rating the acceptance of the treatment as high and 98% of participants indicating satisfaction with the treatment. These findings align with previous research demonstrating high acceptance and satisfaction rates of telehealth psychotherapy for patients with personality disorders [71]. From the participants’ perspective, useability ratings of the telehealth format for delivering DBT were also high, with 69%-83% indicating high usability. Interestingly, participants’ useability ratings significantly increased throughout the treatment, potentially indicating that those with initially low useability ratings may have dropped out of treatment. Unfortunately, our sample size was insufficient to robustly analyze useability as a predictor of therapy dropout during telehealth treatment. Future studies should explore potential predictors for treatment success related to the usability of the telehealth format. In contrast, therapists' perception of telehealth format usability was notably lower, with only half of the therapists (45%-53%) indicating high usability for delivering psychotherapy via telehealth. This discrepancy may be attributed to the greater adjustments required by therapists, transitioning from in-person to telehealth psychotherapy delivery. This finding is consistent with prior research identifying various barriers, challenges, and concerns among therapists delivering DBT via telehealth [20]. Thus, future research should explore strategies to enhance therapists' usability experiences during telehealth treatment. We would also argue that telehealth psychotherapy need not replace in-person psychotherapy, but rather can be used as a complement for clients in need. Both modalities offer valuable options for delivering effective and accessible treatment. Future studies should consider how to tailor treatment delivery format to individual preferences and circumstances.

### Limitations

There are some limitations that should be considered when interpreting the study’s results. First, the exclusion of patients with comorbid PTSD limits the representativeness of the studied population, particularly its applicability and generalizability to all patients with BPD. Therefore, we caution against generalizing these results to patients with BPD and comorbid PTSD. Second, the absence of a control group directly comparing telehealth and in-person conditions within the same trial and cohort impedes drawing inferential conclusions regarding the relative efficacy of telehealth versus in-person DBT. Lastly, the small sample size of 39 clients did not allow us to analyze potential moderator effects on treatment outcomes or dropout rates. Given the limitations of this pilot and feasibility study, future research should include RCTs with larger sample sizes to directly compare telehealth and in-person DBT treatments.

### Conclusions

Our findings provide a first indication that telehealth DBT for BPD appears clinically efficacious, safe, feasible, and well accepted. However, the dropout rate was higher compared to other studies investigating in-person delivered DBT. Future research should compare the efficacy of telehealth DBT with in-person formats in RCTs. Overall, telehealth-delivered DBT offers a potentially effective alternative to in-person DBT, enhancing treatment accessibility and overcoming barriers to treatment engagement. This knowledge may be particularly useful for increasing access to treatment for those in rural areas or low- and middle-income countries where traveling distances to specialized DBT practitioners or treatment programs may be a large barrier.

## Data Availability

All data generated or analyzed during this study are included in this article. Further inquiries can be directed to the corresponding author and will be made available on request.

## Appendix

Multimedia Appendix 1 Acceptance, satisfaction, useability, and therapeutic alliance measures of clients during telehealth DBT. DBT: dialectical behavior therapy.

Multimedia Appendix 2 Acceptance, satisfaction, useability, and therapeutic alliance measures of therapists during telehealth DBT. DBT: dialectical behavior therapy.

## Abbreviations

AIM: Acceptability of Intervention Measure
ATP: according to protocol
BPD: borderline personality disorder
BSL-23: Borderline Symptom List-23
CSQ-8: Client Satisfaction Questionnaire
DBT: dialectical behavior therapy
DSHI: Deliberate Self-Harm Inventory
DSM-5: *Diagnostic and Statistical Manual of Mental Disorders* (Fifth Edition)
DSS: Dissociative Symptom Scale
ITT: intention-to-treat
NSSI: nonsuicidal self-injury
PTSD: posttraumatic stress disorder
RCT: randomized controlled trial
RR: risk ratio
SAE: severe adverse event
UTAUT: unified theory of acceptance and use of technology
WAI: Working Alliance Inventory
