# Supplementary figures and images for: Acceptance, Safety, and Effect Sizes in Online Dialectical Behavior Therapy for Borderline Personality Disorder: Interventional Pilot Study
Source: JMIR Form Res. 2025 Jan 14;9:e66181. doi: 10.2196/66181 (PMC11775487; doi:10.2196/66181)

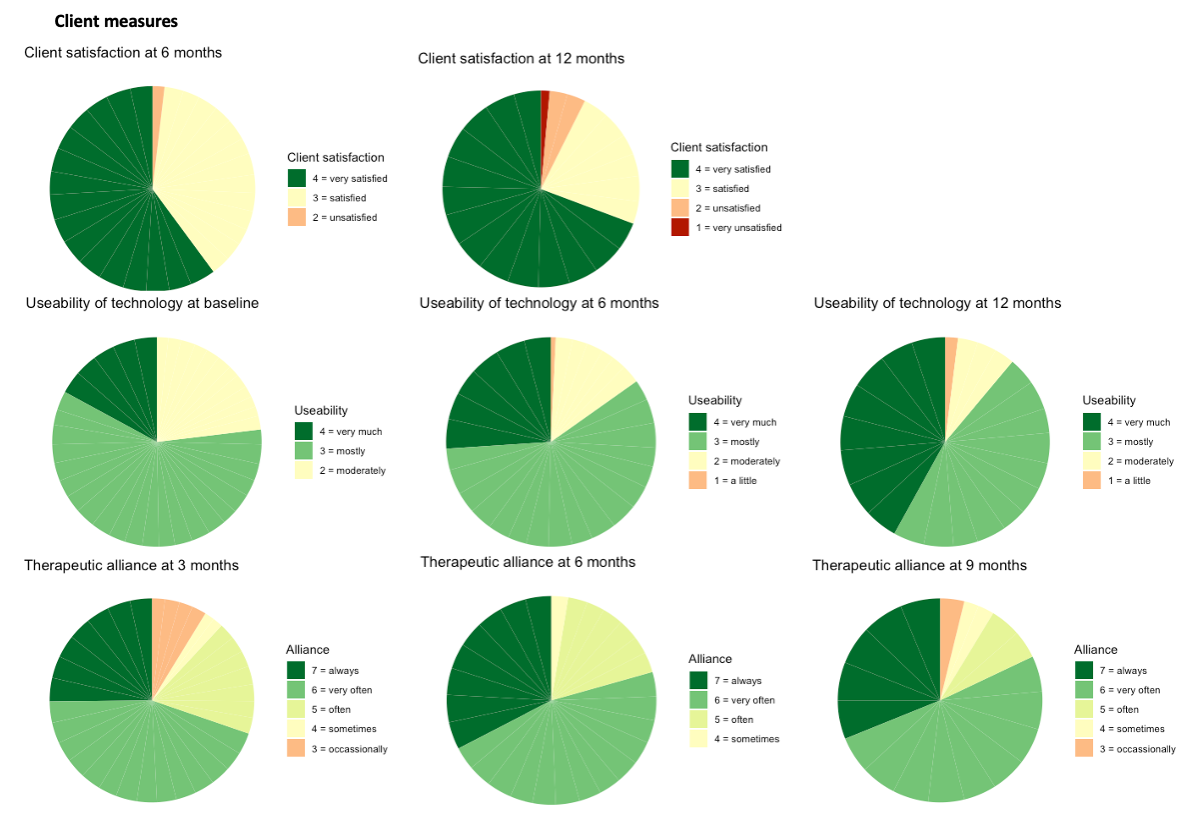

Supplement: Multimedia Appendix 1 [file formative_v9i1e66181_app1.png]

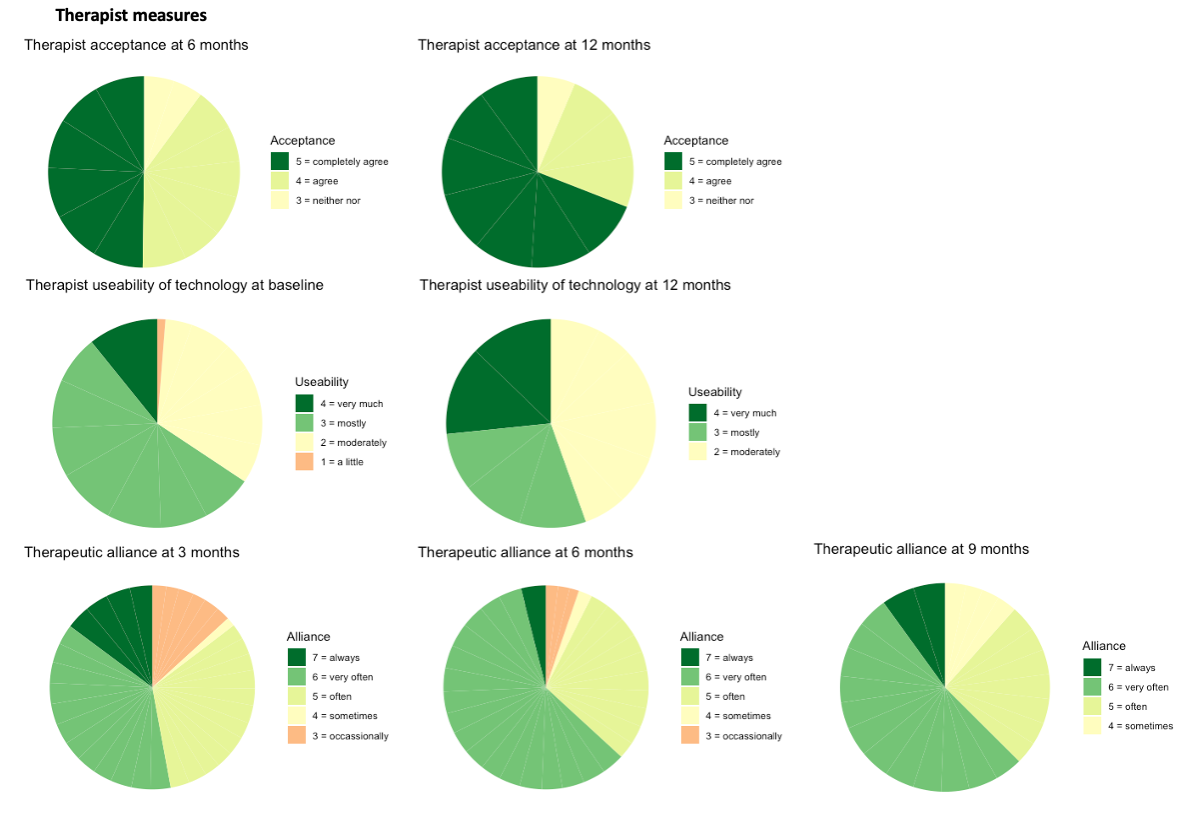

Supplement: Multimedia Appendix 2 [file formative_v9i1e66181_app2.png]
